# Protein S Negatively Regulates Neural Stem Cell Self-Renewal through Bmi-1 Signaling

**DOI:** 10.3389/fnmol.2017.00124

**Published:** 2017-05-02

**Authors:** Katya Zelentsova-Levytskyi, Ziv Talmi, Ghada Abboud-Jarrous, Tal Capucha, Tamar Sapir, Tal Burstyn-Cohen

**Affiliations:** ^1^Faculty of Dental Medicine, Institute for Dental Sciences, Hebrew University-HadassahJerusalem, Israel; ^2^Department of Molecular Genetics, Weizmann Institute of ScienceRehovot, Israel

**Keywords:** Protein S, PROS1, self-renewal, neurogenesis, neural stem cells, Bmi-1

## Abstract

Revealing the molecular mechanisms underlying neural stem cell self-renewal is a major goal toward understanding adult brain homeostasis. The self-renewing potential of neural stem and progenitor cells (NSPCs) must be tightly regulated to maintain brain homeostasis. We recently reported the expression of Protein S (PROS1) in adult hippocampal NSPCs, and revealed its role in regulation of NSPC quiescence and neuronal differentiation. Here, we investigate the effect of PROS1 on NSPC self-renewal and show that genetic ablation of *Pros1* in neural progenitors increased NSPC self-renewal by 50%. Mechanistically, we identified the upregulation of the polycomb complex protein Bmi-1 and repression of its downstream effectors p16^Ink4a^ and p19^Arf^ to promote NSPC self-renewal in *Pros1*-ablated cells. Rescuing *Pros1* expression restores normal levels of Bmi-1 signaling, and reverts the proliferation and enhanced self-renewal phenotypes observed in *Pros1*-deleted cells. Our study identifies PROS1 as a novel negative regulator of NSPC self-renewal. We conclude PROS1 is instructive for NSPC differentiation by negatively regulating Bmi-1 signaling in adult and embryonic neural stem cells.

## Introduction

Regulation of Neural stem and progenitor cell (NSPC) proliferation and self-renewal is key for normal development and adult brain homeostasis. Hippocampal NSPCs give rise to newborn neurons through the process of neurogenesis, contributing to learning and memory (Gould et al., 1999; Shors et al., 2001; Deng et al., 2010; Murai et al., 2014). The pool of NSPCs is maintained throughout life due to self-renewing divisions, while differentiating daughter cells give rise to neurons and astrocytic functional cells (Seri et al., 2004; Ming and Song, 2005; Suh et al., 2007; Bonaguidi et al., 2011). In the hippocampus, radial neural stem cells (NSCs; type 1 cells) morphologically span the hippocampal granular layer with their cell soma located at the subgranular zone (SGZ) of the hippocampal dentate gyrus, and their branched dendritic arbor facing the hippocampal molecular layer. Radial NSCs give rise to neurons, astrocytes, and additional NSCs, and are thus multipotent and self-renewing (Suh et al., 2007; Bonaguidi et al., 2011). Molecularly, they express Nestin, Sox2, GFAP, and Vimentin, and are seldom labeled with 5-Bromo-2′deoxyuridine (BrdU), comprising the replication-quiescent population of neural progenitors; QNPs (Garcia et al., 2004; Seri et al., 2004; Suh et al., 2007). Upon activation, radial NSCs undergo morphological and molecular changes: they retract their dendritic arbor and main shaft, adopting a rounded, horizontal morphology. The cytoplasm of horizontal (type 2) cells is restricted to the SGZ, and molecularly GFAP expression is downregulated, while Nestin and Sox2 are maintained (Suh et al., 2007; Bonaguidi et al., 2011; Encinas et al., 2011). Type 2 NSPCs frequently incorporate BrdU, thus are Amplifying Neural Progenitors-ANPs (Suh et al., 2007). Following proliferation, ANPs undergo a series of asymmetric divisions to self-replicate in a process of self-renewal, and to differentiate mostly into neurons through an intermediate phase of doublecortin-expressing (DCX^+^) neuronal precursors through neurogenesis. Hippocampal NSCs differentiate into glial and neuronal cells, indicating their multipotency (Seri et al., 2004; Suh et al., 2007; Bonaguidi et al., 2011)-a capacity maintained when grown in culture.

The potential to conduct self-renewing cell divisions allows for the ongoing maintenance of a NSPC pool for future cell renewal, while cell divisions yielding differentiated progeny including both neurons and astrocytes provide a continuous source of functional cells. The balance between self-renewal and differentiation must be tightly regulated, as failure to self-renew will lead to stem cell depletion.

The polycomb complex protein Bmi-1 (B cell-specific Moloney murine leukemia virus integration site 1) is a key regulator of adult stem cell self-renewal in the nervous (Molofsky et al., 2003, 2005) and hematopoietic (Lessard and Sauvageau, 2003) systems. Bmi-1 contributes to stem cell self-renewal by negatively regulating the transcription of the cell cycle inhibitors p16^Ink4a^ and p19^Arf^ (Molofsky et al., 2005). However, the precise mechanism and molecular determinants underlying adult NSPC self-renewal remain largely unknown.

The TAM family of receptor tyrosine kinases, comprising of TYRO3, AXL, and MERTK and their cognate ligands Growth-Arrest-Specific 6 (GAS6) and Protein S (PROS1) are expressed by embryonic and adult NSPCs (Wang et al., 2011; Gely-Pernot et al., 2012; Ji et al., 2014; Zelentsova et al., 2016). The simultaneous inactivation of all three TAM receptors in adult NSPCs induced their apoptotic cell death *in vitro* (Ji et al., 2014), however neither the roles nor the mechanisms by which TAMs function in NSPC biology are fully understood.

Increased proliferation of NSPC often results in the rapid depletion of the NSC pool as observed following the inactivation of Notch1 (Ables et al., 2010) or its downstream effector RBPJ (Ehm et al., 2010), BMPR1A (Mira et al., 2010), ApoE (Yang et al., 2011), CHD7 (Jones et al., 2015), or VEFG (Kirby et al., 2015). However, unexpectedly and contrary to such cases, the increased NSPC proliferation in *Pros1*-cKO adult mice did not result in the depletion of the NSPC pool. Instead, we observed persistent, active NSPC proliferation up to 1 year-old aged mice (Zelentsova et al., 2016), indicating the unique uncoupling between NSPC proliferation, increased neurogenesis and subsequent depletion of the NSPC pool. This irregular phenomenon raised the hypothesis that loss of *Pros1* expression in NSPCs results in their increased proliferation concomitant with an increase in self-renewal. Here, we report a novel role for *Pros1* in regulating NSPC self-renewal.

PROS1 is a secreted glycoprotein, expressed by various cells in numerous tissues (Stitt et al., 1995; Lemke, 2013). Ligand-mediated activation of TAM signaling instigates numerous key intracellular signaling pathways leading to various cellular outcomes necessary for tissue homeostasis (Lemke and Burstyn-Cohen, 2010; Lemke, 2013). Inactivation of TAM receptor signaling culminates in major organ defects, including apoptosis of blood vessels, male sterility, blindness, and autoimmune disease (Lu et al., 1999; Lu and Lemke, 2001; Prasad et al., 2006; Burstyn-Cohen et al., 2009; Rothlin et al., 2015). We recently showed that PROS1 functions as a TAM ligand *in vivo*, necessary to maintain a healthy retina (Burstyn-Cohen et al., 2012). PROS1 is also a potent blood anticoagulant (Ten Kate and van der Meer, 2008), however its role as a TAM agonist is independent of its function in maintaining blood fluidity. In the nervous system, PROS1 is expressed by neurons, astrocytes, and microglia (He et al., 1995; Wang et al., 2011; Gely-Pernot et al., 2012; Butovsky et al., 2014; Zelentsova et al., 2016).

To test whether PROS1 plays a role in NSPC self-renewal, we measured the self-renewing divisions and NSPC numbers following knockout of *Pros1* in nestin-expressing cells (*Pros1*-cKO). We find that increased NSPC proliferation does not result in increased cell death and that compared to control *Pros1*^*fl*/*fl*^ littermates, subventricular and hippocampal SGZ NSPCs are more numerous in *Pros1*-cKO mice. NSPCs isolated from both cortical E14.5 and adult *Pros1*-cKO SGZ undergo increased self-renewal. *Pros1*-cKO mice have upregulated Bmi-1 levels, and decreased levels of the cell cycle inhibitors p16^Ink4a^ and p19^Arf^. Rescuing *Pros1* expression in cKO NSPCs restored proliferation rates, Bmi-1 levels and self-renewal rates. Taken together, our results indicate that PROS1 regulates the NSPC pool through balancing NSPC proliferation and self-renewal through Bmi-1-signaling.

## Materials and methods

### Mice

All mice were maintained in the Hebrew University-Hadassah vivarium, following institutional IACU guidelines. Pros1^fl/fl^ (Burstyn-Cohen et al., 2009), Nestin-Cre (Tronche et al., 1999), and Nestin-GFP (Mignone et al., 2004) mice were previously described. *In vivo* experiments on adult mice were performed on mice 2–4 months of age.

### Immunohistochemistry

Mice were deeply anesthetized by intraperitoneal Keatmine/Xylazine solution, and transcardially perfused as previously described (Burstyn-Cohen et al., 2012). The brains isolated, immersion-fixed overnight at 4°C in 4% PFA in PBS, cryopreserved for 24 h at 4°C in 30% sucrose/PBS, and embedded in OCT (Tissue-Tek). Fifty micron-thick cryosections were collected into PBS and stored in Methanol at −20°C until used. For immunostaining, sections were washed three times in PBS, blocked (5% FBS, 0.1% Triton X-100 in PBS) 1 h at room temperature, followed by primary antibody incubation overnight at 4°C. Next, after PBS washes, sections were incubated with a fluorophore-coupled secondary antibody in blocking buffer for 1 h at room temperature, Hoechst counterstained, and mounted with Fluoromount G (Southern Biotech). TUNEL staining was performed using the *In situ* Cell death Detection Kit (Roche) according to manufacturer's instructions. Images were taken with an Olympus BX51 fluorescent microscope mounted with a DP72 camera, or with a Nikon C2 confocal LASER scanning microscope.

For *in vivo* proliferation studies, intraperitoneal injections of bromodeoxyuridine (BrdU, Sigma, 50 mg/kg) were given for 3 days, and animals were sacrificed 24 h after last BrdU injection. To detect BrdU, cryosections were incubated in 50% formamide for 2 h at 65°C, rinsed in 2XSSC for 15 min/ room temperature (RT). DNA was denatured in 2N HCl for 30 min at 37°C and neutralized with 0.1 M Boric Acid, followed by three washes in 1X TBS (5M NaCl, 1M Tris in DDW) and blocked in blocking buffer (3% FBS, 0.25% Triton X-100 in TBS) for 1 h at RT, followed by incubation with primary rat anti-BrdU for 72 h at 4°C. Next day, the sections were washed three times in 1XTBS and re-blocked in blocking buffer for 15 min at RT, followed by incubation with fluorescently-conjugated secondary antibody in blocking buffer for 1 h at RT. Sections were documented after nuclei staining, as described above.

For BrdU incorporation in neurospheres, cultures were incubated with BrdU (10 μM) for 4 h, fixed in 100% ice-cold methanol for 20 min on ice, and washed three times in 1XPBS. DNA was denatured in 2N HCl for 10 min at 37°C and neutralized with 0.1M Boric Acid for 10 min at RT, followed by three washes in 1X PBS and blocked in the above blocking buffer for 1 h at RT. Finally, spheres were incubated with anti-BrdU antibody and processed as described above. All antibodies used in this study are listed in Supplementary Table 1.

### NSC isolation from embryonic day 14.5

Staged embryos (morning of vaginal plug was considered E0.5) were isolated, forebrains dissected, and the meningeal membranes and blood vessels removed. Telenchephalic cortices were dissected and processed individually to maintain genetic homogeneity. Tail DNA was taken for genotyping. Tissues were mechanically dissociated and Trypsin digested (0.125%, Biological Industries) for 15 min at 37°C, then neutralized for 2 min with trypsin inhibitor (1.25 mg/ml, Sigma), and DNAse I-digested 600 U/ml, Sigma) for 2 min at RT. Serum-free media (DMEM/F12; 100 U/ml penicillin; 0.1 mg/ml streptomycin; 2 mM L-Glutamine) was added and cells were centrifuged at 1,200 rpm for 10 min (acceleration = 9, deceleration = 1). Pelleted cells were resuspended in growth medium (serum free medium as above, supplemented with 1x B27 (Gibco) and hbFGF (10 ng/ml, PeproTech Asia) and seeded (300,000 cells/ml) in 10 cm cell culture plates in growth medium. Fresh hFGF was added daily. Neurospheres were grown for 2 days *in vitro* (2 div), then mechanically dissociated and reseeded at a clonal density of 300,000 cells/ml to generate secondary neurospheres. For self-renewal assays, isolated NSCs (embryonic or adult-generated) were plated at clonal density of 2,500–5,000 cells per well in 2 ml growth medium as above supplemented with 20 ng/ml EGF (PeproTech Asia). All cultures were maintained at 37°C in 5% CO_2_. To measure self-renewal, individual neurospheres were mechanically dissociated, then replated at a clonal density of 2,500–5,000 cells per 3.5 cm diameter well. Primary and secondary neurospheres were counted after 3 div. To evaluate subpopulations, neurospheres were acutely dissociated in Accutase solution (1X, Millipore, Temecula), plated on pre-coated poly-Orinithine (0.5 mg/ml, Sigma) sterile cover slides, allowed to adhere for 2–3 h and fixed immediately in 4% PFA to prevent differentiation. Cells were further processed for immunofluorescence as described above. PROS1 was purchased from Enzyme Research Laboratories (South Bend, IN). To determine PROS1 dosage we performed preliminary experiments using different PROS1 concentrations. Thirty-five nanomoles per liters of PROS1 was found to be effective, without apparent toxicity effects on NSCs.

### Electroporation of NSCs and constructs

Plasmid constructs were transfected into NSCs by electroporation, using the NEPA21 electroporation system (Nepa Gene, Japan), according to manufacturer's instructions. Mouse *Pros1* cDNA was excised from the pcDNA6-mPros1 vector (a gift from Greg Lemke, Prasad et al., 2006) using EcoR1 and Xho1, and cloned using the same restriction sites into the pCAGGS expression vector harboring an IRES-nuclear GFP reporter. shBmi-1 and control sh-irr vectors were a gift from Dinorah Friedman-Morvinski.

### NSC isolation from adult dentate gyrus

Eight to twelve weeks old mice were deeply anesthetized and sacrificed. The brains isolated and placed in ice-cold HBSS (Biological Industries) containing 10 mM Hepes (Biological Industries) until dissected midsagitally; the dentate gyri isolated and kept in HBSS/10 mM Hepes on ice. All solutions were used ice-cold, unless otherwise stated. The tissue was enzymatically dissociated in 5 ml of Solution 1 [5.4 mg/ml D-Glucose (Sigma), 15 mM HEPES (Biological Industries) 680 ng/ml Trypsin (Sigma) and 700 ng/ml hyaluronidase (Sigma) in 1 × HBSS] for 30 min at 37°C, with gentle trituration after 15 min. Cells were mixed with 5 ml Solution 3 (40 mg/ml BSA (Sigma), 20 mM HEPES, 1 × EBSS (Biological Industries) passed through a 70 μm strainer, and centrifuged at 1,300 rpm for 5 min. The pellet was resuspended in 10 ml Solution 2 [308 mg/ml Sucrose (Sigma) in 0.5 × HBSS], and centrifuged at 2,000 rpm for 10 min, then resuspended in 2 ml Solution 3, and carefully loaded onto 12 ml of Solution 3, and pelleted by centrifugation at 1,500 rpm for 7 min. Cellular debris was removed by centrifugation; the cells resuspended in 15 ml medium in neurosphere growth medium [DMEM:F12, 100 U/ml penicillin; 0.1 mg/ml streptomycin; 2 mM L-Glutamine, 1 × B27 w/o vitamin A (Gibco), 10 mM Hepes]. EGF (20 ng/ml) and FGF2 (10 ng/ml) was supplemented daily. After 3 div, primary neurosphere cultures were resuspended in 15 ml growth medium, cellular debris was removed by centrifugation; the cells resuspended in growth medium and replated. Growth medium was replaced every 3 div, neurospheres were taken for analysis at 10 div. For self-renewal assays, at 3 div cells were treated as above to rid of cellular debris, and resuspended at 2,500 cells/1.5 cm diameter dish (24 well) thus are considered secondary neurospheres. Four days later, cells were collected, dissociated. and replated at the same density to generate tertiary neurospheres.

### Quantitative real-time PCR (qPCR)

Total RNA was extracted from DG (carefully dissected and cleaned from adjacent tissue under a dissecting stereoscope) or cultured neurospheres with TRIZOL (Sigma). cDNA was synthesized (qScript cDNA synthesis kit, Quanta Biosciences). Real-Time reactions were performed in triplicate using KAPA SYBR FAST qPCR Kit (2X) following manufacture's recommendation on a CFX96 Real Time PCR system (Bio-Rad). Expression levels were calculated relative to *Gapdh*, using the ΔΔ threshold cycle (Ct) method. Primer sequences are provided in Supplementary Table 2. Quantitative RT-qPCR determinations were done in at least three biological replicates with similar results.

### Western blotting

Protein was extracted from DG or neurospheres in lysis buffer (50 mM Tris pH7.5, 150 mM NaCl, 1%Triron X-100, 0.5% NP-40, 0.1% SDS, 0.5 mM EDTA) supplemented with protease inhibitor cocktail (Sigma), incubated on ice for 30 min, and cellular debris removed by centrifugation. Protein concentration was determined by the BCA method (Thermo Scientific). Samples (5–20 μg) were run on 10% acrylamide gels and transferred to PVDF membranes (Millipore). ECL signal was detected using a ChemiDoc™ MP Image System (Bio-Rad), and band quantification was done using ImageJ.

### FACS analysis

Cells were prepared as previously described (Moussaud and Draheim, 2010). Briefly, adult mice were transcardially perfused with cold 1X PBS. DG were isolated and a single cell suspension was prepared by enzymatic digestion [20 units/ml papain (Worthington) in 116 mM NaCl, 5. NS, no statistical significance 4 mM KCl, 26 mM NaHCO_3_, 1 mM NaH_2_PO_4_, 1.5 mM CaCl_2_, 1 mM MgSO_4_, 0.5 mM EDTA, 25 mM glucose, 1 mM cysteine] for 60–90 min at 37°C, 5% CO_2_. After incubation, fetal bovine serum (FBS) was added to quench enzymatic activity (20% final concentration in HBSS, Biological Industries). Cells were collected by centrifugation (200 g,7 min, RT), resuspended and incubated for 5 min at room temperature in 0.5 mg/ml DNAse I (Sigma), passed through a 70 micron cell strainer (BD bioscience), and centrifuged (200 g, 7 min). Cells were then separated by a gradient density centrifugation using 0.9 M sucrose in HBSS and centrifuged at 200 g for 20 min with slow acceleration and no brake, resuspended in 2% FBS in PBS, and incubated with primary antibody for 30 min on ice. Cells were twice washed in 2% FBS and incubated with secondary antibody for 30 min on ice. Samples were washed in 2% FBS, filtered through 70 micron mesh and read by an LSR II FACS (BD Biosciences, San Jose, CA) and analyzed using FlowJo software (Ashland, OR).

### Statistics

Data represent mean ± SEM of experiments. At least three independent experiments were performed per assay. Significance was determined using a Student's *t*-test, considering *P* < 0.001 as extremely significant (^***^), *P* < 0.01 as highly significant (^**^) and *P* < 0.05 as statistically significant (^*^).

## Results

### Increased numbers of NSPCs in the hippocampal SGZ following *Pros1* deletion

**Figure 1:**
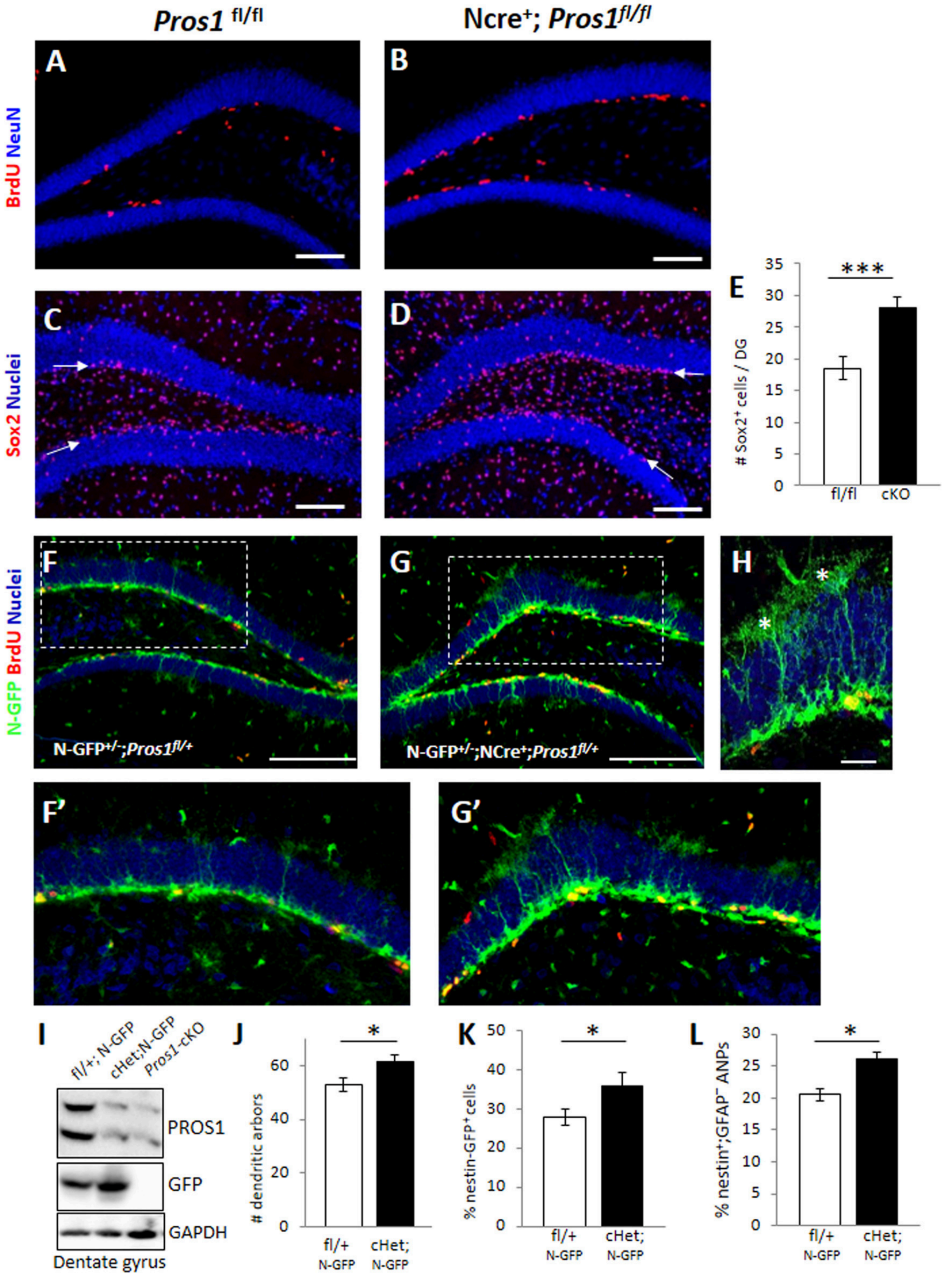
**Deletion of *Pros1* in neural cells leads to superfluous hippocampal neural stem cells. (A,B)** Confocal images of adult hippocampus showing BrdU-positive cells (red) in *Pros1*^*fl*/*fl*^ (control, **A**) and *Pros1*-cKO **(B)** adult mice. The dentate gyrus is delineated by NeuN immune-reactivity (blue). Mice were injected with BrdU for three consecutive days and analyzed 24 h after the last BrdU injection. **(C–E)** Increased Sox2^+^ cells in the SGZ of *Pros1-*cKO mice. Confocal images of Sox2-labeled cells (red) in control **(C)** and *Pros1*-cKO **(D)** adult SGZ. Nuclei are stained with Hoechst (blue). Arrows point to the SGZ. **(E)** Quantification of sox2^+^ cells in the SGZ). Bars represent the mean values ± SEM from six mice per genotype. ^***^P < 0.0001. Scale bar: **(A–D)** = 100 μm. **(F–H)** Representative confocal images of Nestin-GFP cells (green) and BrdU immunoreactivity (red) in control **(F**,**F**′**)** and Pros1-cHet **(G**,**G**′**)** adult SGZ. **(F**′**,G**′**)** are close up views of the boxed areas in **(F,G)**, respectively. **(H)** Quiescent (radial) NSCs are easily detected as nestin-GFP^+^ cells spanning the granule cell with elaborate dendritic arbors extending into the molecular layer (asterisks). Scale bar: **(F,G)** = 100 μm; **(H)** = 20 μM. **(I)** Western blot analysis of hippocampal cell extracts from the indicated mice. Blots were reacted for PROS1 (top), GFP (center), and GAPDH (bottom) as a loading control. A blot representative of four independent experiments is shown. **(J)** Morphology-based quantification of the radial glia-like quiescent NSCs dendritic arbors from mice with the indicated genotypes. Dendritic arbors were defined as GFP^+^ ramifications extending from the granular layer into the molecular layer (asterisks in **L**). Mean values and standard errors were derived from five individual mice per genetic group. ^*^P = 0.04. **(K,L)** Quantification of nestin-GFP-positive cells by FACS, representing the total NSC population (**K**; ^*^P = 0.04) and of nestin-GFP^+^;GFAP^−^ cells representing the horizontal NSC sub-population (**L**; ^*^P = 0.017). Mean values and standard errors were derived from five individual mice per genetic group.

We recently reported that the conditional deletion of *Pros1* in NSPCs led to a 52 and 57% increase in SVZ and SGZ NSPC proliferation, respectively (Zelentsova et al., 2016). Similar results depicting the increased NSPC proliferation in the SGZ are presented in Figures 1A,B. Moreover, increased NSPC proliferation was observed in culture from NSPCs isolated from either E14.5 cortices or adult SGZ NSPCs, demonstrating a similar role for *Pros1* in NSPC proliferation regardless of their developmental stage or topographic location. Unexpectedly, the increased NSPC proliferation did not result in depletion of the NSPC pool. Rather, NSPC proliferation continued throughout adulthood, indicating functional NSPC proliferation in aged mice (Zelentsova et al., 2016).

We set to explore the consequences of augmented NSPC proliferation on the NSPC population. For this, we first assessed the numbers of Sox2^+^ cells located in the SGZ by immunostaining and found a 50% increase in the numbers of Sox2^+^ cells confined to the SGZ of *Pros1*-cKO adult mice, compared to control littermates (Figures 1C–E), suggesting increased numbers of NSPCs. To verify these Sox2^+^ cells are indeed NSPCs, we crossed our mice to the nestin-GFP reporter mice (Mignone et al., 2004) allowing the visualization and quantification of bona-fide NSPC based on nestin-GFP expression, and on morphological features distinguishing radial glial-like NSCs from horizontal NSPCs. We found a significant increase in the overall numbers of both radial glial-like QNP and horizontal NSPCs in Nestin-Cre^+^; *Pros1*^*fl*/+^; Nes-GFP (cHet; N-GFP) mice, also seen by nestin-GFP immunoreactivity in western blots of DG extracts (Figures 1F–I). Glial-like radial NSCs were scored based on both nestin-GFP expression and the presence of radial morphology with an extensive dendritic tree (Figures 1H,J). The highly-proliferative horizontal NSPCs also express nestin-GFP, but their accurate scoring was not possible by IHC due to over-crowdedness of cells (Figures 1F–H). We therefore took advantage of GFAP downregulation as NSCs transit from the radial to horizontal mode (Filippov et al., 2003; Seri et al., 2004), while sustaining expression of nestin-GFP (Encinas et al., 2011), and FACS- analyzed single cell suspensions of dentate gyri. Radial quiescent GFAP^+^; Nestin-GFP^+^ and horizontal, proliferating GFAP^−^; nestin-GFP^+^ cells were scored. In agreement with our IHC analysis, we observed a significantly higher percentage of GFP-positive cells in *Pros1*-cHet mice than in control mice (Figure 1K and Supplementary Figure 1A). Additionally, the GFAP^−^; GFP^+^ population was also increased compared to control mice (Figure 1L and Supplementary Figure 1B), indicating an overall increase in both radial/quiescent and horizontal/amplifying NSPCs. Noteably, the deletion of a single *Pros1* allele was sufficient to generate this phenotype. Taken together, these data demonstrate increased numbers of NSPCs following ablation of PROS1.

### Effect of *Pros1* deletion on NSPC numbers is cell-autonomous

**Figure 2:**
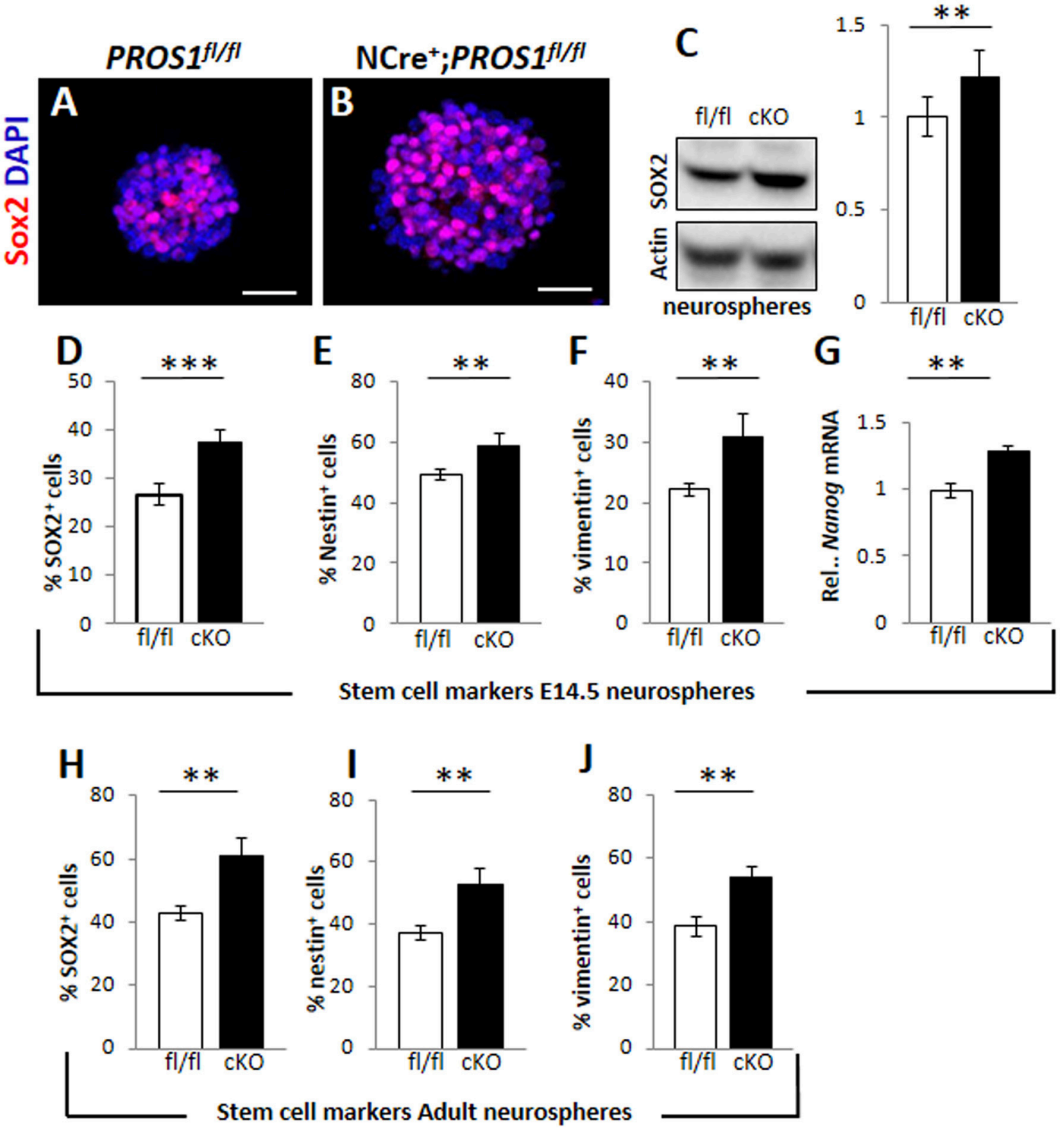
**Increased cell numbers expressing neural stem cell markers in neurospheres derived from *Pros1*-cKO NSCs. (A,B)** Representative confocal images of Sox2 immunoreactivity in neurospheres generated from E14.5 control **(A)** and *Pros1*-cKO **(B)** NSCs. Scale bar: 50 μm. **(C)** Western blot analysis of Sox2 expression in culture-grown neurospheres. Whole cell extracts were isolated from spheres and subject to analysis. Actin reactivity serves as a loading control. Band intensities were quantified using NIH image J software. A blot representative of at least three different experiments is shown. Quantification of graphs represent mean values and standard errors from at least three independent experiments. ^**^P = 0.008. **(D–G)** Quantification of cells expressing the neural stem cell protein markers Sox2 (**D**; *P* = 0.00066), Nestin (**E**; *P* = 0.01), vimentin (**F**; *P* = 0.01), and RT-qPCR relative expression levels of nanog (**G**; *P* = 0.01) in control and *Pros1*-cKO neurospheres generated from E14.5 embryos. Mean values and standard errors were derived from five individual experiments. A minimum of three and up to five embryos per genotype were pooled in each experiment. **(H–J)** Quantification of cells expressing the neural stem cell protein markers Sox2 (**H**; *P* = 0.0012), Nestin (**I**; *P* = 0.0038), vimentin (**J**; *P* = 0.0016) in control and *Pros1*-cKO neurospheres generated from adult SGZ NSCs. Pooled cells from 3 to 4 mice per genetic group were used for each experiment. Mean values and standard errors were derived from four individual experiments. ^***^P ≤ 0.001; ^**^P ≤ 0.05.

*Pros1*-cKO adult brains contain many cells in which *Pros1* expression is retained, including astrocytes, endothelial cells, and microglia (Burstyn-Cohen et al., 2009; Butovsky et al., 2014; Zelentsova et al., 2016), in which *Nestin*-Cre is not active, and may thus contribute to these effects. Investigating NSC self-renewal in *Pros1* null mice is impossible due to embryonic lethality caused by hypercoagulability and vascular dysgenesis (Burstyn-Cohen et al., 2009). We therefore tested the effects of PROS1 deficiency in isolated NSPC-derived neurosphere cultures, thus excluding any possible PROS1 complementation by *Pros1*-expressing niche cells. Wild type neurospheres isolated from E14.5 embryonic NSPCs grown for 10 days expressed PROS1, which co-localized with all three NSPC markers tested: Sox2, nestin, and vimentin (Supplementary Figure 2), indicating that PROS1 expression in NSPCs is maintained under culture conditions. Neurospheres generated from *Pros1*-cKO NSPCs E14.5 brains had significantly more NSPCs, as indicated by the number of cells positive for the Nestin, Sox2, vimentin and nanog (*P* < 0.01 for nestin and nanog; *P* < 0.001 for Sox2 and vimentin; *N* = 5; Figures 2A–G and Supplementary Figure 2). Spheres generated from adult SGZ NSPCs also had significantly more cells positive for NSPC markers (*P* < 0.004 for nestin and Sox2; *P* < 0.002 for vimentin; *N* = 4; Figures 2H–J). Similarly, elevated nestin, decreased DCX and increased S100b immune-reactivity were observed in cultured neurospheres (Supplementary Figure 3). These results are consistent with our *in vivo* observations indicating elevated numbers of NSPCs following *Pros1* deletion, and indicate that increased NSPC numbers is a capacity maintained when grown in culture, and is common to NSPCs generated from both embryonic and adult brains.

**Figure 3:**
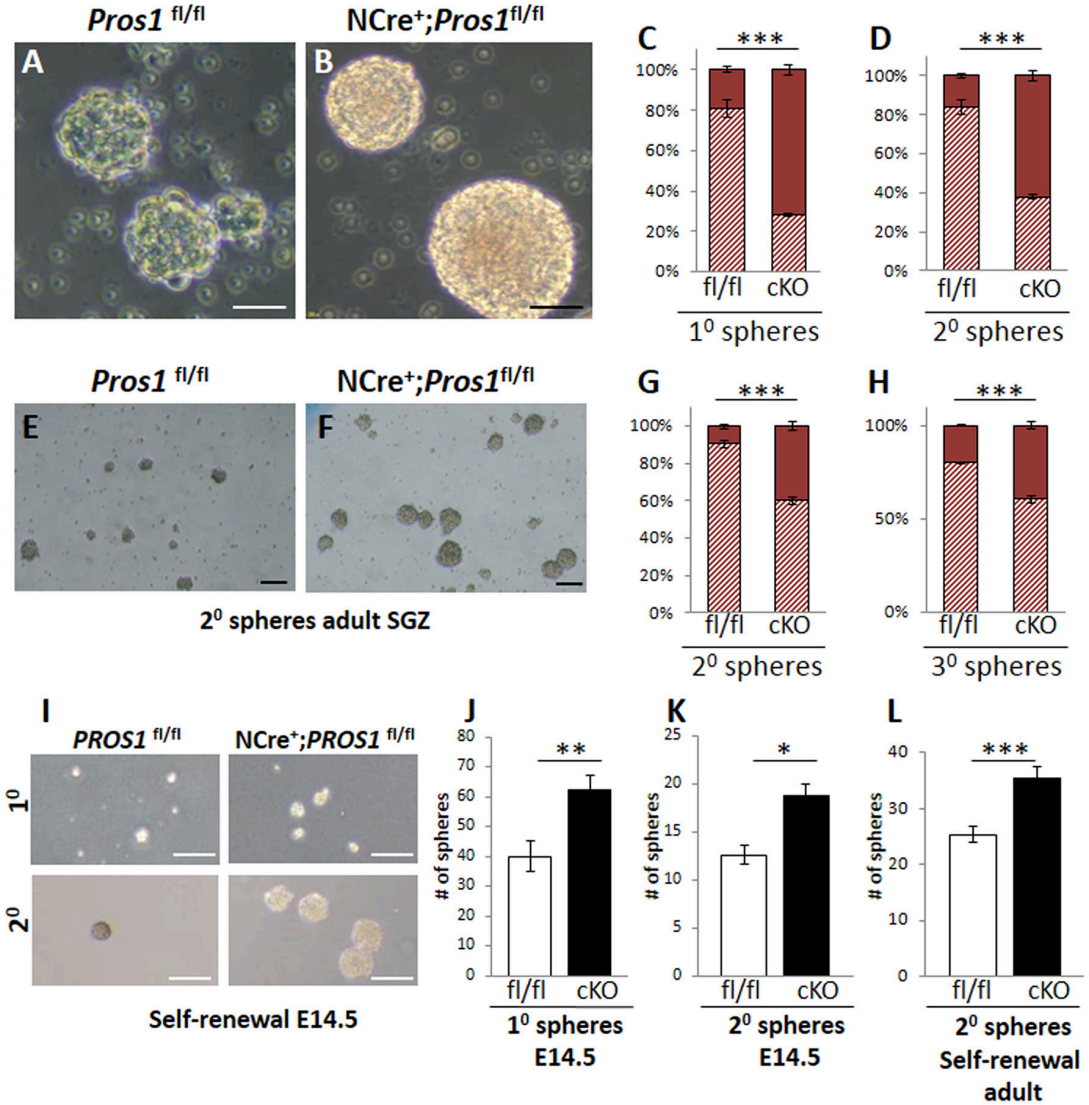
**Increased self-renewal following *Pros1*-deletion. (A–D)** Representative phase-contrast images of neurospheres generated from control **(A)** and *Pros1*-cKO **(B)** E14.5 NSCs and grown in culture for 4 days. Scale bar = 20 μM. **(C–D)** Quantification of primary **(C)** and secondary **(D)** neurospheres with diameter smaller or larger than 35 microns (hashed bars and solid bars, respectively) from control and *Pros1*-cKO NSCs. Mean values and standard errors were derived from 4 individual experiments. A minimum of three and up to five embryos per genotype were pooled in each experiment. ^***^P < 0.0001. Statistics are derived from measuring at least 100 neurospheres per experiment. **(E–H)** Representative phase-contrast images of secondary neurospheres generated from control **(E)** and *Pros1*-cKO **(F)** adult SGZ NSCs and grown in culture for 14 days. Scale bar = 50 μM. **(G–H)** Quantification of secondary **(G)** and tertiary **(H)** neurospheres with diameter smaller or larger than 35 microns (hashed bars and solid bars, respectively) from control and *Pros1*-cKO adult SGZ NSCs. Pooled cells from 3 to 4 mice per genetic group were used for each experiment. Mean values and standard errors were derived from four individual experiments. ^***^P < 0.0001. Statistics are derived from measuring at least 50 neurospheres per experiment. **(I–L)** Self-renewal assays from control and *Pros1*-cKO NSCs isolated from E14.5 cortices **(I–K)** and adult SGZ **(L)**. **(I)** Representative images depicting self-renewal capacity of primary (top panels) and secondary (lower panels) neurospheres. Scale bar: 200 μm. Quantification of the number of primary (**J**; *P* = 0.002) and secondary (**K**; *P* = 0.02) neurospheres generated from 2,500 cells, after 10 days in culture. Mean values and standard errors were derived from four individual experiments. A minimum of three and up to five embryos per genotype were pooled in each experiment. **(L)** Quantification of the number of secondary neurospheres generated from 2,500 adult-SGZ hippocampal NSCs, after 10 days in culture. *P* = 0.0006. Pooled cells from 3 to 4 mice per genotype were used for each experiment. Mean values and standard errors were derived from four individual experiments. At least 60 E14.5 and 30 adult-derived neurospheres were counted per experiment. ^***^P < 0.001; ^**^P < 0.01; ^*^P < 0.05.

Another feature of *Pros1*-cKO NSPCs cultured from E14.5 brains is that they yielded significantly larger neurospheres than those generated from control littermates. *Pros1*-cKO spheres appeared with a more dense morphology, including a distinct core, whereas individual cells were still apparent in control spheres, resembling immature spheres (Figures 3A,B). Primary neurospheres were dissociated and grown to generate secondary spheres. Both primary and secondary spheres from *Pros1*-cKO embryos were significantly larger than control spheres (Figures 3C,D), demonstrating that increased sphere size is inherent to NSPCs, and that neither NSPC numbers nor sphere size depend upon factors provided by niche cells. To understand whether the increased neurosphere size was also a feature shared by adult hippocampal NSPCs, we isolated, cultured, and assayed neurospheres generated from adult SGZ NSCs. As for E14.5 spheres, and in line with their increased NSPC proliferation, adult-generated spheres from *Pros1*-cKO mice produced significantly larger secondary and tertiary spheres than adult control NSPCs (Figures 3E–H). Taken together, these results show that PROS1 functions to maintain NSPC numbers in a cell-autonomous manner, and that this feature is maintained upon passages in culture. Additionally, *Pros1* functions similarly in NSPCs generated from embryonic and adult SGZ, suggesting a common role in regulating NSPC homeostasis in development and adulthood.

### *Pros1* deletion does not affect NSPC viability

In the process of adult neurogenesis, only a small subset of newborn neuroblasts survive to maturity, while most newborn cells undergo apoptotic death in the first 4 days of their life (Sierra et al., 2010). The remaining neuroblasts mature within 4 weeks, during which they extend dendrites, migrate into the subgranular zone and finally synapse and integrate within the pre-existing neuronal network, contributing to hippocampal functions such as learning and memory (van Praag et al., 1999; Deng et al., 2010; Murai et al., 2014). To understand whether the elevated numbers of NSPC observed in *Pros1*-cKO may be a result of increased neuronal cell death, we assessed cell death by the Terminal deoxynucleotidyl transferase dUTP nick end labeling (TUNEL) assay. We found control and *Pros1*-cKO DG had similar numbers of TUNEL-positive and cleaved caspase 3-positive (C-Casp3) cell counts (Supplementary Figures 4A–F). Similarly, comparable levels of apoptotic cells were measured in control and *Pros1*-cKO cultured neurospheres by TUNEL and C-Caspase 3 from embryonic and adult-derived NSPCs (Supplementary Figures 4G–M) indicating that loss of *Pros1* does not enhance apoptotic cell death, or delay the clearance of newborn cells. Taken together, enlarged neurosphere size, and lack of increased cell death, all indicate the viability of *Pros1*-cKO newborn cells.

### Enhanced self-renewal of NSCs following *Pros1* deletion

The generation of larger-sized neurospheres could stem from either increased NSPC proliferation, increased NSPC self-renewal, or both. The increased NSPC proliferation following *Pros1* ablation was recently reported (Zelentsova et al., 2016), however the direct impact of *Pros1* ablation on regulation of NSPC self-renewal was not previously investigated. Most NSPCs in the adult DG are self-renewing and multipotent (Suh et al., 2007; Bonaguidi et al., 2011). Maintaining the delicate balance between self-renewal and differentiation is critical to brain homeostasis, as it preserves a constant pool of NSPCs for future tissue renewal, while generating the brain's functional cells at the same time. Given the increased number of cells expressing NSPC markers in *Pros1*-cKO SGZ (Figures 1C–L) and in isolated NSPCs (Figures 2A–J) we hypothesized that loss of PROS1 would affect the self-renewal capacity of NSPCs. We next performed a direct self-renewal assay which revealed that embryonic *Pros1*-cKO NSPCs have a significantly higher capacity to generate primary and secondary neurospheres, as is also the case for adult-generated SGZ NSPCs (Figures 3I–L), demonstrating that elimination of *Pros1* expression in NSCs increases stem cell self-renewal in both embryonic and adult neurospheres grown *in vitro*. Importantly, increased numbers of NSPCs does not result from elevated levels or cell death, as TUNEL and cleaved-Caspase 3 assays yielded similar values for *Pros1*-cKO and control cells both *in vivo* and *in vitro* (Supplementary Figure 4). Taken together with our observation of increased radial and horizontal NSPCs *in vivo* (Figures 1F–L), with more numerous Nestin^+^, Sox2^+^, and vimentin^+^ cells in isolated neurospheres (Figures 2A–J) we conclude that deletion of *Pros1* results in increased neural stem cell self-renewal.

### Pros1 inhibits self-renewal through repression of Bmi-1

**Figure 4:**
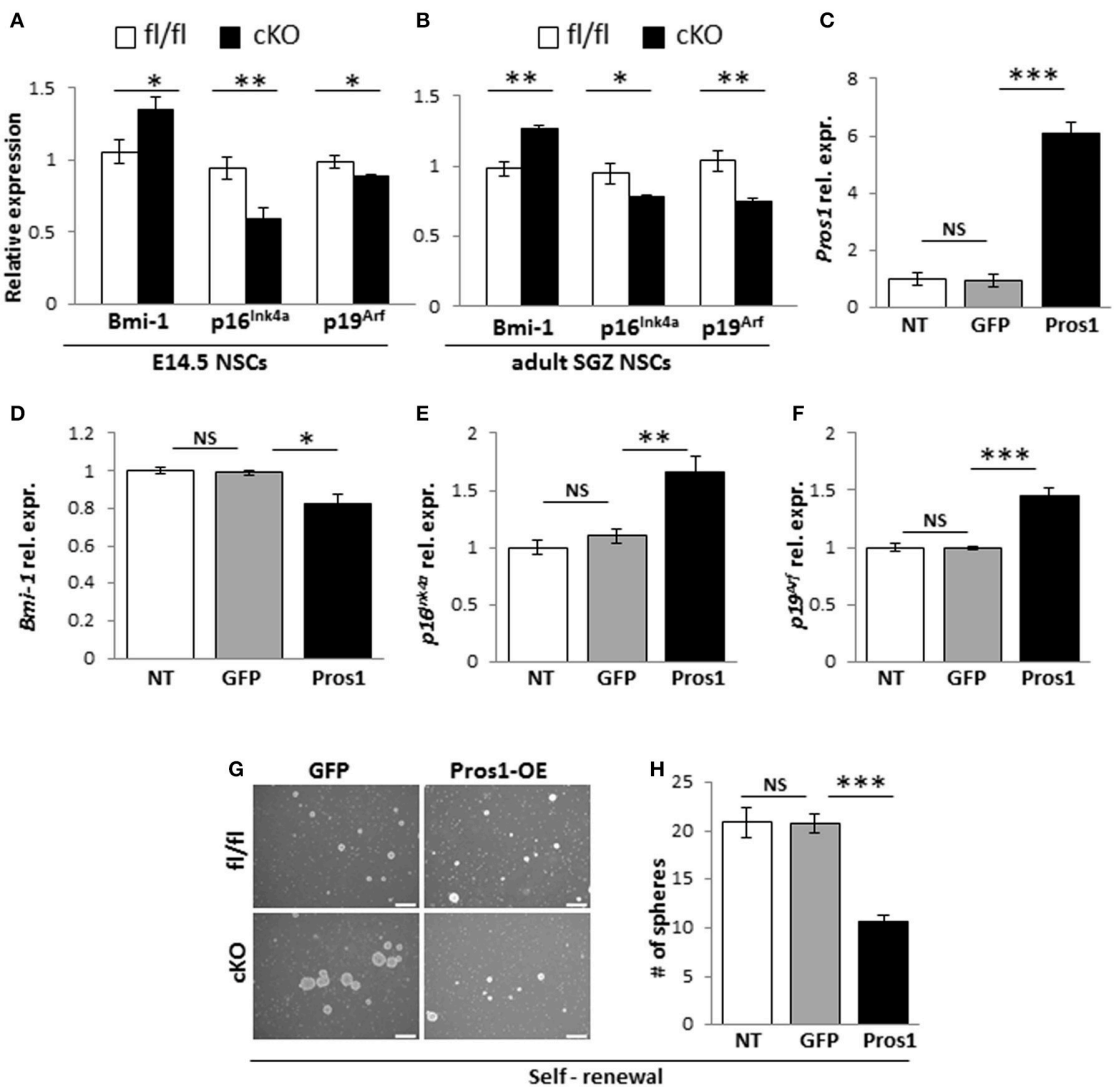
***Pros1*** **suppresses NSC self-renewal through Bmi-1 inhibition. (A,B)** RT-qPCR relative expression levels of Bmi-1, and the Bmi-1 downstream genes p16^Ink4a^ and p19^Arf^ in neurospheres derived from E14.5 **(A)** and adult SGZ **(B)** NSPCs from control (white bars) and cKO (black bars) cells. Mean values ± SEM were derived from 4 individual experiments. A minimum of three and up to five embryos per genotype or 3–4 adult mice were pooled in each experiment. **(C–F)** Rescuing *Pros1* expression in cKO NSPCs restores Bmi-1 signaling. **(C)** *Pros1* expression was determined by qRT-PCR from neurospheres derived from *Pros1*-cKO NSPCs from non-treated control (NT, white bar), GFP-electroporated control (gray bar), and *Pros1*-electroporated (black bar) NSPCs. ^***^P < 0.001; NS, no statistical significance. All cells were allowed to grow for 3 days following electroporation. Graphs represent mean values ± from four individual experiments, with at least two embryos from each genotype pooled in each experiment. **(D–F)** Rescuing *Pros1* expression downregulates Bmi-1 levels (**D**; *P* = 0.03), along with upregulation of p16^Ink4a^ (**E**; *P* = 0.013) and p19^Arf^ (**F**; *P* = 0.001) in cKO NSPCs treated with a Pros1-expressing vector (black bars). Non-treated NSCs (NT, white bar), or cells electroporated with a GFP control vector (gray bar) were not affected. NS, no statistical significance. Graphs represent mean values ± from four individual experiments. At least two embryos from each genotype pooled in each experiment. **(G,H)** Phenotypic rescue following PROS1 rescue in cKO NSPCs. Phase contrast **(G,H)** quantification of self-renewal rates performed on non-treated (NT, white bar), control-electroporated (GFP, gray bar) or *Pros1*-electroporated (Pros1, black bar) cells. ^***^P < 0.0001; NS, no statistical significance. At least 60 neurospheres per genotype were scored for statistical evaluation. Graphs represent mean values ± from four individual experiments. ^***^P < 0.001; ^**^P < 0.01; ^*^P < 0.05.

Bmi-1 is a negative transcriptional regulator of p16^Ink4a^ and p19^Arf^ in NSCs (Molofsky et al., 2003, 2005). Bmi-1 deficiency in NSCs leads to reduced self-renewal (Molofsky et al., 2003), while its overexpression resulted in larger neurospheres with more self-renewing divisions (He et al., 2009). Given its role in NSC self-renewal; and the increased self-renewal observed in *Pros1*-deficient NSCs, we hypothesized the increased self-renewal observed in *Pros1*-cKO NSCs may be mediated through *Bmi*-1. We found elevated *Bmi-1* expression in *Pros1*-cKO neurospheres derived from embryonic NSPCs (Figure 4A). Both p16^Ink4a^ and p19^Arf^ expression were significantly inhibited in *Pros1*-cKO spheres (Figure 4A), consistent with Bmi-1 being a transcriptional repressor for both genes. Akin to embryonic NSPCs, *Bmi-1* expression was significantly increased in adult cKO NSPCs (Figure 4B). These results indicate *Pros1* similarly affects the expression of Bmi-1 pathway genes in both embryonic and adult NSPCs.

To determine whether Bmi-1 expression and self-renewal were directly related to PROS1 levels, we performed a rescue experiment by electroporating a GFP-tagged *Pros1* expression vector into *Pros1*-cKO neural stem cells, restoring *Pros1* expression in cKO NSPCs to control levels (Figure 4C). Compared to treatment-naïve and control-treated (GFP-encoding vector) cells, rescuing *Pros1* expression in cKO NSPCs repressed Bmi-1 expression (Figure 4D), and elevated the transcript levels of p16^Ink4a^ and p19^Arf^, consistent with Bmi-1 being a repressor for both genes (Figures 4E,F). Phenotypically, the rescue of *Pros1* expression in cKO NSCs reversed their increased self-renewal levels (Figures 4G,H).

**Figure 5:**
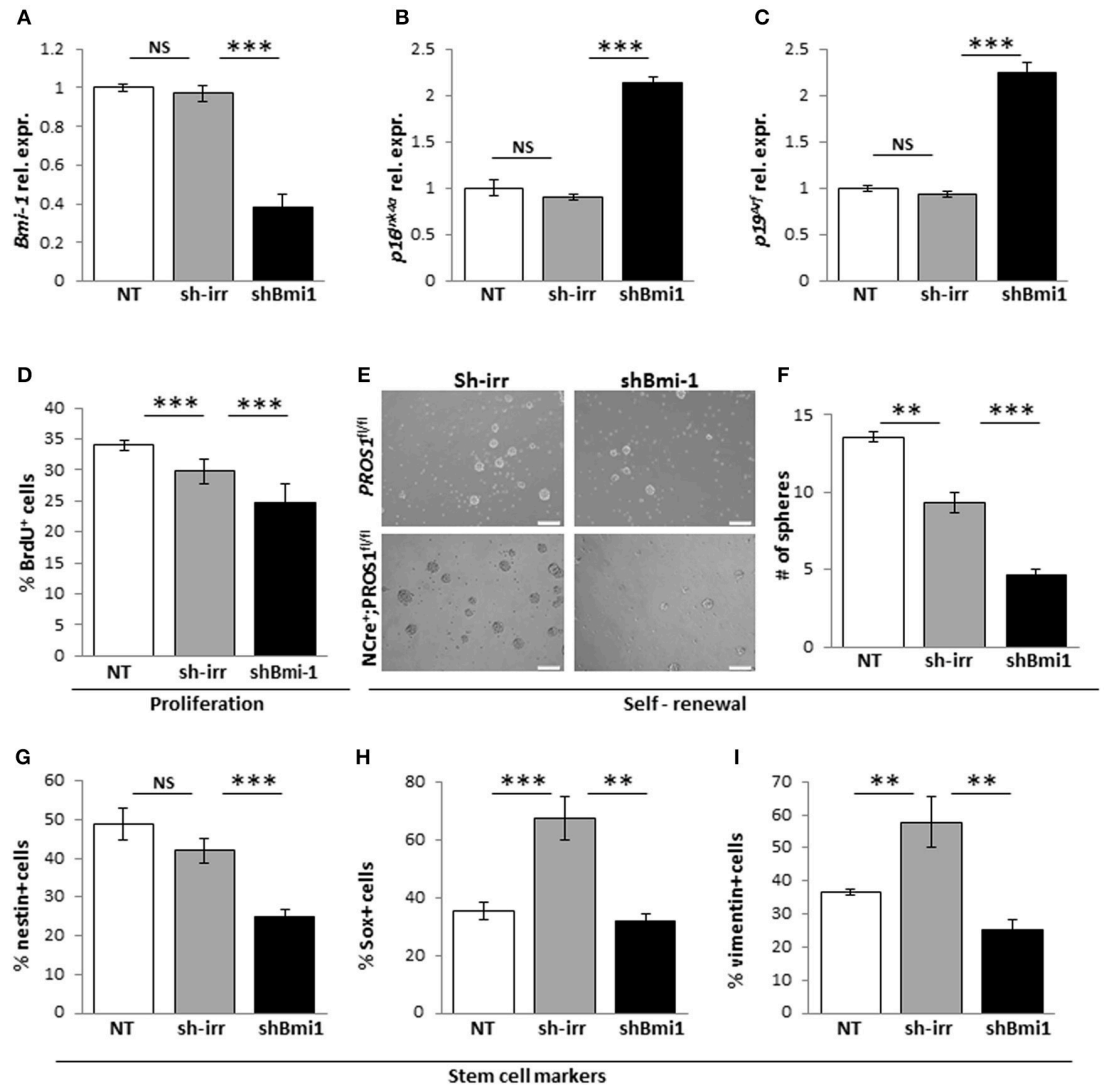
**Knockdown of Bmi-1 in *Pros1*-cKO NSCs restores Bmi-1 signaling and self-renewal**. RT-qPCR relative expression levels of Bmi-1 **(A)** p16^Ink4a^ **(B)** and p19^Arf^ **(C)** in neurospheres derived from *Pros1*-cKO NSPCs following Bmi-1 knockdown. ^***^P < 0.001; NS, no statistical significance. Non-treated NSPCs (NT, white bar), or cells electroporated with a control sh vector (sh-irr, gray bar) were not affected. Cells electroporated with sh-Bmi-1 are presented by the black bars. Graphs represent mean values ± from four individual experiments. At least two embryos from each genotype were pooled in each experiment. **(D–I)** Bmi-1 knockdown in cKO NSPCs suppresses their increased proliferation and self-renewal. Proliferation **(D)** was measured by BrdU incorporation (10 μM added to the culture medium 4 h prior to fixation), and self-renewal **(E,F)** was measured by the number of neurospheres generated from 2,500 NSPCs. Neurospheres were documented by phase-contrast microscopy **(E)** and quantified **(F)**. ^***^P < 0.001. At least 60 neurospheres per genotype were scored for statistical evaluation. Graphs represent mean values ± from four individual experiments. At least two embryos from each genotype were pooled in each experiment. **(G–I)** Phenotypic rescue of NSCs markers following shBmi-1 in cKO NSPCs. Scoring the percent of nestin+ **(G)**, Sox2+ **(H)**, and vimentin+ **(I)** cells in non-treated, sh-irr and shBmi-1 treated cells. ^***^P < 0.001; ^**^P < 0.01. Graphs represent mean values ± from four independent experiments. At least two embryos from each genotype were pooled in each experiment.

Next, to verify that the elevated Bmi-1 levels in *Pros1*-cKO NSCs were directly related to increased self-renewal in our cells, we inhibited the elevated Bmi-1 levels in *Pros1*-cKO NSCs by means of sh-RNA (Figure 5A). This lead to a sharp increase in p16^Ink4a^ and p19^Arf^ expression (Figures 5B,C), reversing the increased proliferation and self-renewal phenotypes (Figures 5D–F). The decreased self-renewal following Bmi-1 knockdown in cKO NSCs also corresponded to restoration of stem cell numbers, as indicated by immune-detection of Nestin, Sox2, and vimentin (Figures 5G–I). Taken together, these results demonstrate that *Pros1* negatively regulates NSPC self-renewal, through inhibition of Bmi-1.

**Figure 6:**
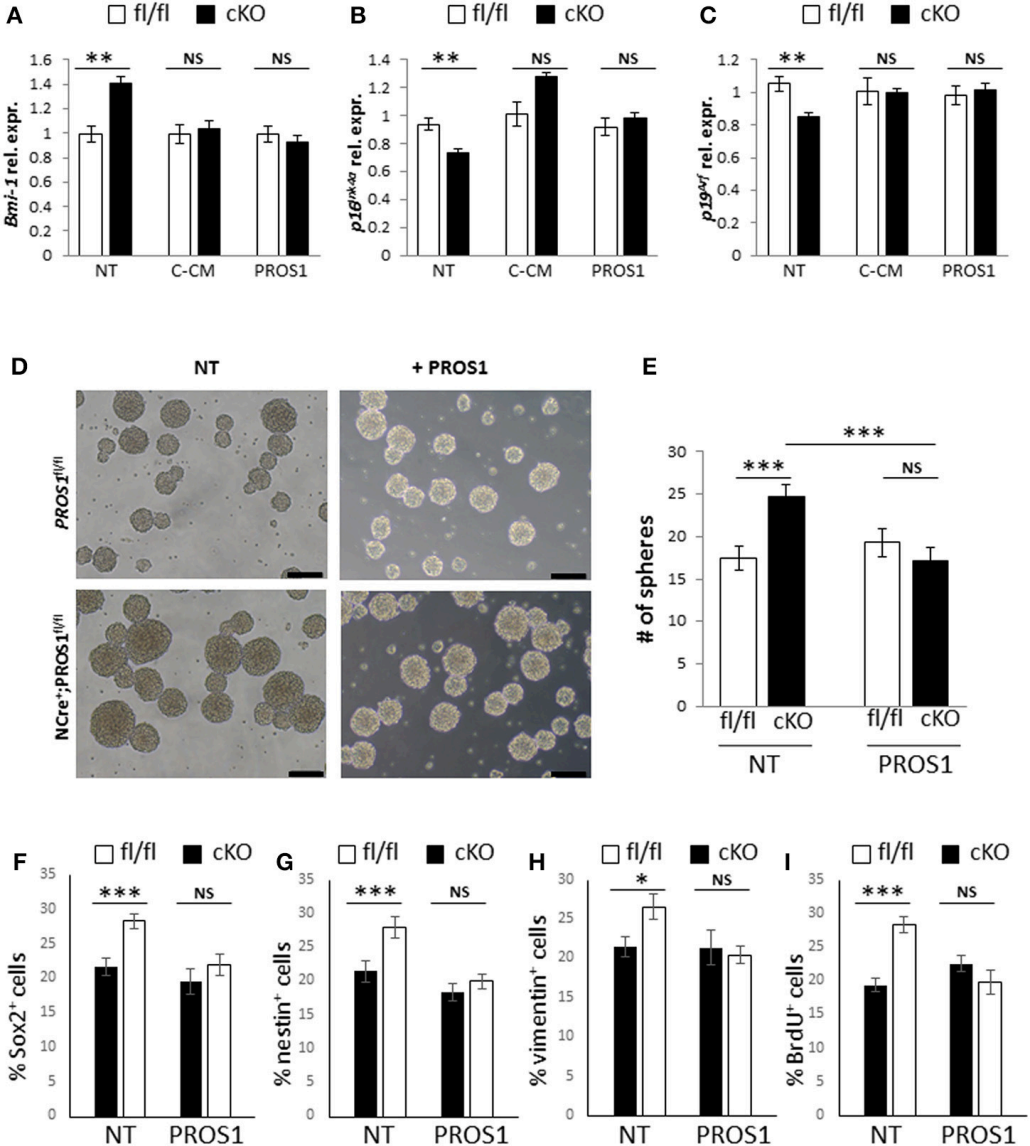
**Both Bmi-1 pathway genes and self-renewal are rescued following addition of exogenous PROS1. (A–C)** Purified PROS1 rescues Bmi-1 pathway gene expression. RT-qPCR relative expression levels of Bmi-1 **(A)** p16^Ink4a^ **(B)** and p19^Arf^ **(C)** in neurospheres derived from *Pros1*-cKO NSPCs that were grown either in their own conditioned medium (NT), following exchange of CM from control cells (C-CM), or grown in medium supplemented with purified PROS1 (35 nmol/Lit). ^**^P < 0.01; NS, no statistical significance. Mean values ± SEM were derived from 4 independent experiments. Statistics are derived from measuring at least 100 neurospheres in each experiment. **(D–I)** Neural stem cell self-renewal in *Pros1*-cKO NSPCs is rescued by purified PROS1. Phase contrast **(D)** and quantification **(E)** of self-renewal following addition or not of purified PROS1 (35 nmol/Lit) from NSPCs derived from control (fl/fl, white bars) or *Pros1*-cKO mice (cKO, black bars). ^***^P < 0.001; NS, no statistical significance. Mean values ± SEM were derived from four independent experiments. Statistics are derived from measuring at least 200 neurospheres per condition in each experiment. Scale bars: 50 μM. Decreased self-renewal by exogenous PROS1 is also evident by the numbers of cells positive for the NSC markers Sox2 **(F)**, nestin **(G)**, vimentin **(H)**, and by decreased proliferation as measured by BrdU incorporation **(I)**. ^***^P < 0.001; ^**^P < 0.01; ^*^P < 0.05; NS, no statistical significance. Mean values ± SEM were derived from four independent experiments.

Finally, to gain insight into the mechanistic action of PROS1, we took advantage of the secretory nature of PROS1 and tested whether NSPC cultures expressing and secreting PROS1 could rescue both Bmi-1 levels and the self-renewal phenotype in *Pros1*-cKO cells. Conditioned medium (CM) from control *Pros1*^*fl*/*fl*^ NSPCs (C-CM) rescued the expression levels of *Bmi-1, p16*^*Ink*4*a*^ and *p19*^*Arf*^ in *Pros1*-cKO NSPCs (Figures 6A–C), bringing them to control levels. Moreover, supplementation of *Pros1*-cKO NSPCs with purified PROS1 protein also reversed *Bmi-1, p16*^*Ink*4*a*^ and *p19*^*Arf*^ expression (Figures 6A–C), highlighting the direct effect of PROS1 on Bmi-1 signaling. The addition of purified PROS1 further rescued both the self-renewal phenotype of *Pros1*-cKO cells bringing them to levels comparable to those of control NSPCs (Figures 6D,E), and the numbers of cells expressing the NSC markers Sox2, nestin, and vimentin (Figures 6F–H). Consistent with lower levels of Bmi-1 (and higher *p16*^*Ink*4*a*^ and *p19*^*Arf*^), cells were also less proliferative, as indicated by reversal of the high incorporation levels of BrdU (Figure 6I). Taken together, our results point to PROS1 as a novel regulator of NSPC homeostasis, regulating the self-renewal capacity of NSPCs through Bmi-1 and its downstream effectors *p16*^*Ink*4*a*^ and *p19*^*Arf*^.

## Discussion

In this study we identify PROS1 as a novel factor regulating NSPC self-renewal. Moreover, its role is conserved between embryonic and adult mouse NSPCs. In the absence of PROS1, self-renewing divisions of NSPCs are significantly enhanced; resulting in increased NSPC numbers. We find that under normal homeostatic conditions, Bmi-1 levels are negatively regulated by PROS1, contributing to the exit from self-renewing divisions. The elevated levels of Bmi-1 present in *Pros1*-cKO NSPCs were rescued by reintroducing a *Pros1*-expressing plasmid or by providing purified protein to *Pros1*-cKO NSPCs. Stem cell numbers were also reversed, resembling control brains. NSPC proliferation is intimately linked to both self-renewal and differentiation. We recently reported the genetic ablation of *Pros1* expression in NSPCs (Zelentsova et al., 2016) using the Nestin-Cre driver, which led to a Notch1-dependent exit from quiescence, leading to increased NSPC proliferation. The progeny of these Nestin-Cre^+^; *Pros1*^*fl*/*fl*^ (cKO) NSPCs developed predominantly into astrocytes, at the expense of neurons, indicating a role for PROS1 not only in NSPC quiescence, but also in the acquisition of a neuronal cell fate (Zelentsova et al., 2016). Unexpectedly, the increased proliferation rates of *Pros1*-depleted NSPCs did not lead to depletion of the NSC pool (Zelentsova et al., 2016). Here, we reveal the role of PROS1 as a negative regulator of NSPC self-renewal, whereas in the absence of PROS1, NSPCs simultaneously present with increased proliferative and self-renewal capacities, generating significantly more nestin+, Sox2+, and vimentin+ NSCs. Enhanced rates of both proliferation and self-renewal is maintained in culture, indicating PROS1 functions cell-autonomously, independent of niche cells. We further identify PROS1 as a negative regulator of Bmi-1 signaling affecting cell-cycle exit and re-entry in NSPCs.

Our findings provide original insight into the link between neural stem cell proliferation and self-renewal. Numerous factors were shown to play a role in maintaining NSC quiescence, including Notch1 signaling components (Breunig et al., 2007; Ables et al., 2010; Ehm et al., 2010; Imayoshi et al., 2010), ApoE4 (Yang et al., 2011), BMPRIA (Mira et al., 2010), and Bmi-1 (Molofsky et al., 2003, 2005). Loss of function of these and other (Mu et al., 2010) stem cell quiescence factors results in increased NSC proliferation which invariably leads to an increased but temporary peak in neurogenesis, rapidly ensued by depletion of the NSC pool. In contrast to these examples, while the loss of PROS1 results in increased NSPC proliferation, the stem cell pool is spared from an accelerated division-coupled depletion due to increased self-renewal and replenishment of NSCs.

Mechanistically, we find that PROS1 functions as a negative regulator of Bmi-1 expression, thus allowing sufficient p16^Ink4^ and p19^ARF^ levels to promote exit from the cell cycle. In NSCs, Bmi-1 deficiency leads to loss of self-renewal (Molofsky et al., 2003, 2005) and its overexpression increases self-renewing cell divisions, coupled with increased neurogenesis *in vitro*, but not *in vivo* (He et al., 2009). Similarly, the increased self-renewing divisions in *Pros1*-deficient NSCs (Figures 3E–L) is coupled with the upregulation of Bmi-1 (Figures 4A,B). These phenotypes are rescued upon re-introduction of *Pros1* expression into NSPCs (Figures 4D–H), and attenuating the elevated Bmi-1 levels in *Pros1*-cKO NSCs rescues the proliferation and self-renewal phenotypes (Figures 5A–I), highlighting NSC plasticity with respect to cell cycle exit and re-entry.

An open question regarding PROS1 function in NSCs is whether PROS1 engages the TAM receptors, which are expressed by embryonic and adult NSCs (Wang et al., 2011; Gely-Pernot et al., 2012; Ji et al., 2014). PROS1 was identified as a TAM ligand (Stitt et al., 1995) but its role as a TAM agonist *in vivo* was controversial for two decades (Godowski et al., 1995; Mark et al., 1996; Nagata et al., 1996; Hall et al., 2001). We and others recently identified PROS1 as a bona fide TAM agonist *in vivo* (Burstyn-Cohen et al., 2009, 2012; Carrera Silva et al., 2013). A recent report shows that neurospheres deleted for all three TAM receptors exhibit slower growth, decreased proliferation and increased apoptosis (Ji et al., 2014), indicating that complete blockade of TAM signaling is destructive, and pharmacological neutralization of both TAM ligands GAS6 and PROS1 lead to increased counts of BrdU+ cells in the adult SVZ (Gely-Pernot et al., 2012). In the present study, our genetic analysis identifies PROS1 as a main regulator of NSC homeostasis, governing NSC proliferation, and self-renewal in a Bmi-1 dependent manner, without affecting NSC viability.

## Conclusion

Our study identifies PROS1 as a key regulator of proliferation and self-renewal in embryonic and adult hippocampal NSPCs. PROS1 expression contributes to NSPC differentiation by negatively regulating Bmi-1 signaling, and functions cell-autonomously, independent of niche cells. Although deletion of PROS1 leads to increased NSC proliferation, these cells show increased self-renewal capacities rather than division-coupled exhaustion of the NSC pool, revealing a novel regulatory element at the NSC proliferation /differentiation choice point.

## Ethics statement

This study was carried out in accordance with the recommendations of IACUC guidelines, ethics committee of the Hebrew University-Hadassah. The protocol was approved by the ethics committee of the Hebrew University-Hadassah.

## Author contributions

KZ-L: Collection and assembly of data, data analysis, and interpretation, final approval of manuscript. ZT: Collection and assembly of data, data analysis and interpretation. GA-J: Collection of data. TC and TS: Experimental design, provision of study material, data analysis, and interpretation. TB-C: Conception and design, financial support, collection and assembly of data, data analysis and interpretation, manuscript writing, and final approval of manuscript.

## Funding

This work was supported by ISF research grant 984/12, ISF equipment grant 1764/12 to TB-C and by the Israel Cancer Research Foundation (ICRF) Research Career Development Award (RDCA) to TB-C. KZ-L received a fellowship from the STEP-GTP in science training.

### Conflict of interest statement

The authors declare that the research was conducted in the absence of any commercial or financial relationships that could be construed as a potential conflict of interest.
