# Supplementary material for: Protein S Negatively Regulates Neural Stem Cell Self-Renewal through Bmi-1 Signaling
Source: Front Mol Neurosci. 2017 May 2;10:124. doi: 10.3389/fnmol.2017.00124 (PMC5411449; doi:10.3389/fnmol.2017.00124)
Supplement: Supplementary file 1 [file DataSheet1.PDF]

Supplementary Material

Protein S Negatively Regulates Neural Stem Cell Self-Renewal through Bmi-1 signaling

Katya Zelentsova, Ziv Talmi, Ghada Abboud-Jarrous, Tamar Sapir, Tal Capucha, and Tal Burstyn-Cohen*

*** Correspondence:** Tal Burstyn-Cohen: talbu@ekmd.huji.ac.il

Supplementary Figures

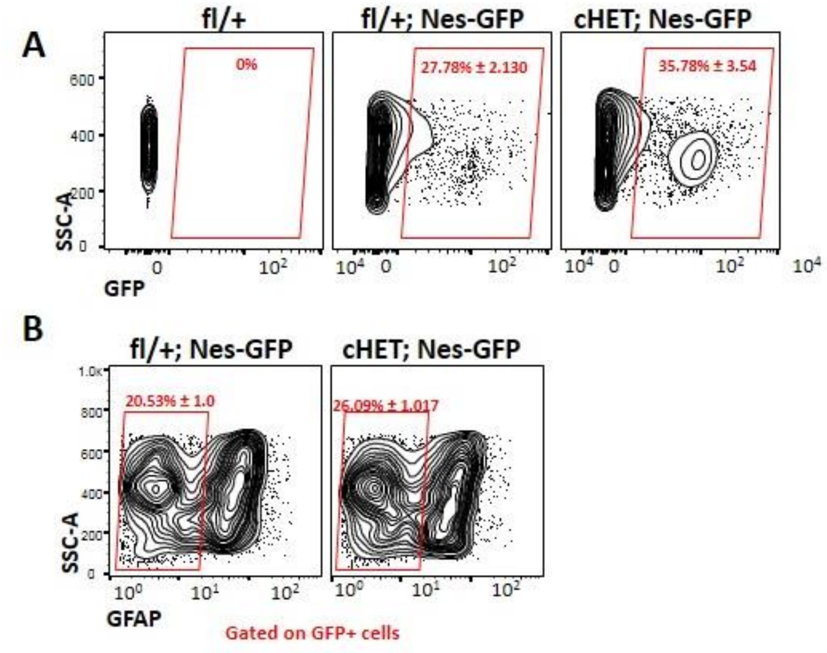

Supplementary Figure 1. Quantification of total Nestin-GFP^{+} neural stem cells and the Nestin-GFP^{+}; GFAP^{-} non-radial NSCs by FACS.

(A) The percent of Nestin-GFP+ cells and the percent of nestin-GFP+; GFAP- cells were identified by flow cytometry in single cell hippocampal suspensions prepared from the indicated mice. Representative FACS plots are shown. GFP expression (X axis) is plotted against the side scatter (granularity, Y axis). GFP signal is not detected in *Pros1*$^{fl/+}$ mice that a do not contain the nestin-GFP transgene (left panel). GFP+ cells, representing both radial and horizontal NSC populations, are more numerous in mice deleted for one *Pros1* allele (cHET; Nes-GFP, right panel) compared to mice expressing *Pros1* from both alleles (*Pros1*$^{fl/+}$; nestin-GFP, central panel). The mean values ± SEM from 5 individual mice is indicated within the gated area, shown in red. (B) Representative FACS plots showing GFP+GFAP- horizontal NSCs. After gating on the GFP+ population, the GFAP-negative cells (delineated within the red framed area) were analyzed. Compared to control mice (left panel), cHET; Nes-GFP mice have increased

numbers of GFP+;GFAP- cells, which represent the horizontal NSC population. The mean values ± SEM from 5 individual mice is indicated within the framed area. Cells were acquired using an LSR II FACS (BD Biosciences, San Jose, CA), and analyzed using FlowJo software.

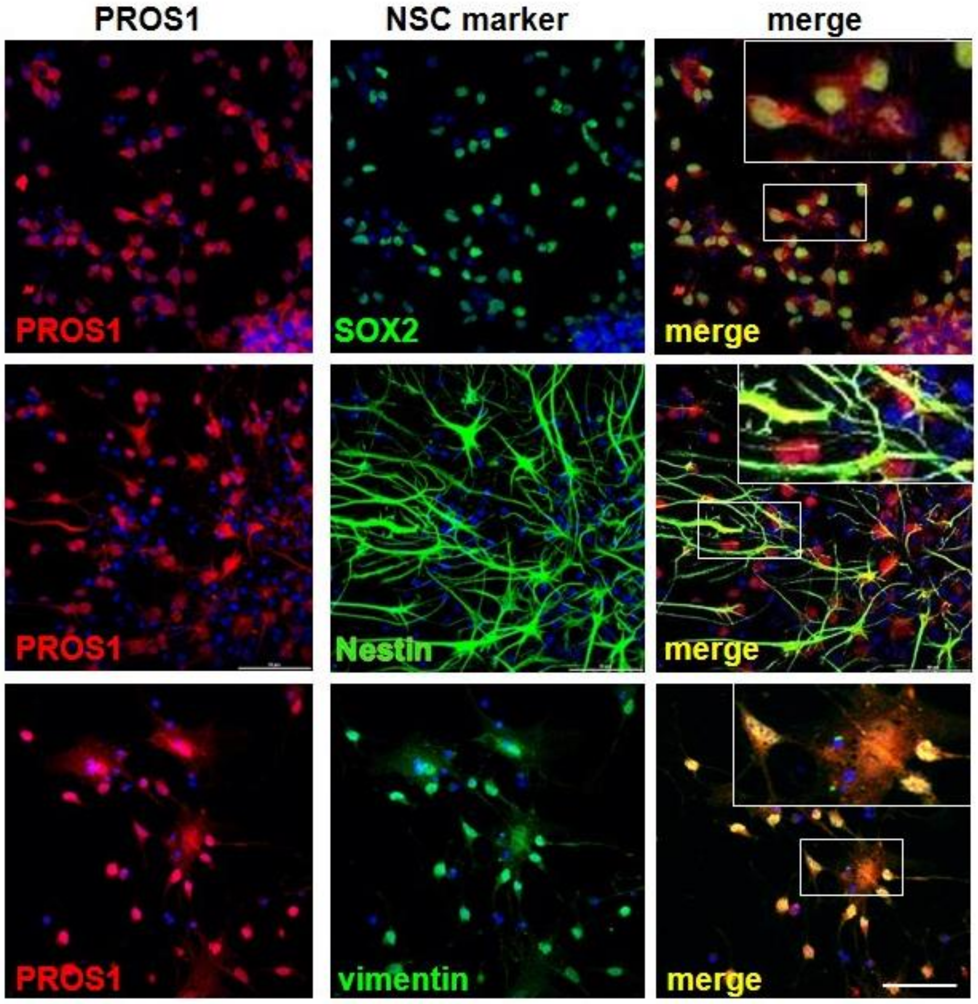

Supplementary Figure 2. Pros1 is expressed in E14.5-derived NSCs grown as neurospheres.

Representative images of NSCs grown in vitro showing PROS1 immunoreactivity (red, left panels) co-localizes with the NSC markers Sox2 (top) Nestin (middle) and vimentin (bottom). Neurospheres were isolated from E14.5 NSCs and cultured for 10 days. Merged images (right panels) include detailed insets. Nuclei are stained with Hoechst (blue). Scale bar: 50 µm.

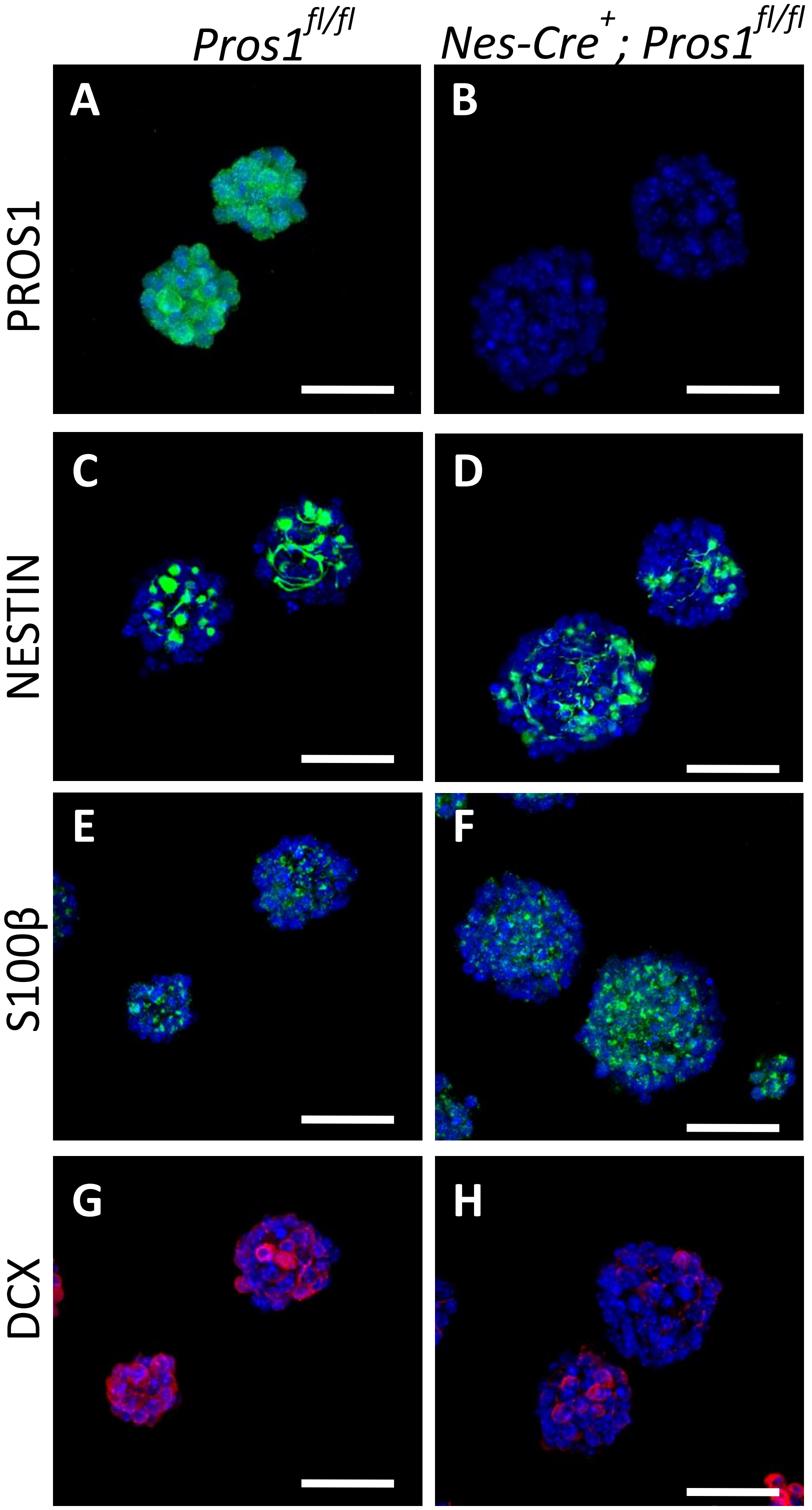

Supplementary figure 3: Additional validation for marker expression in neurospheres.

Whole mount immunohistochemical detection of PROS1 (A, B), Nestin (C, D); S100β (E, F) and DCX (G, H) in neurospheres generated from control (A, C, E, G) and *Pros1*-cKO (B, D, F, H) NSCs. Scale bars: 50 µM.

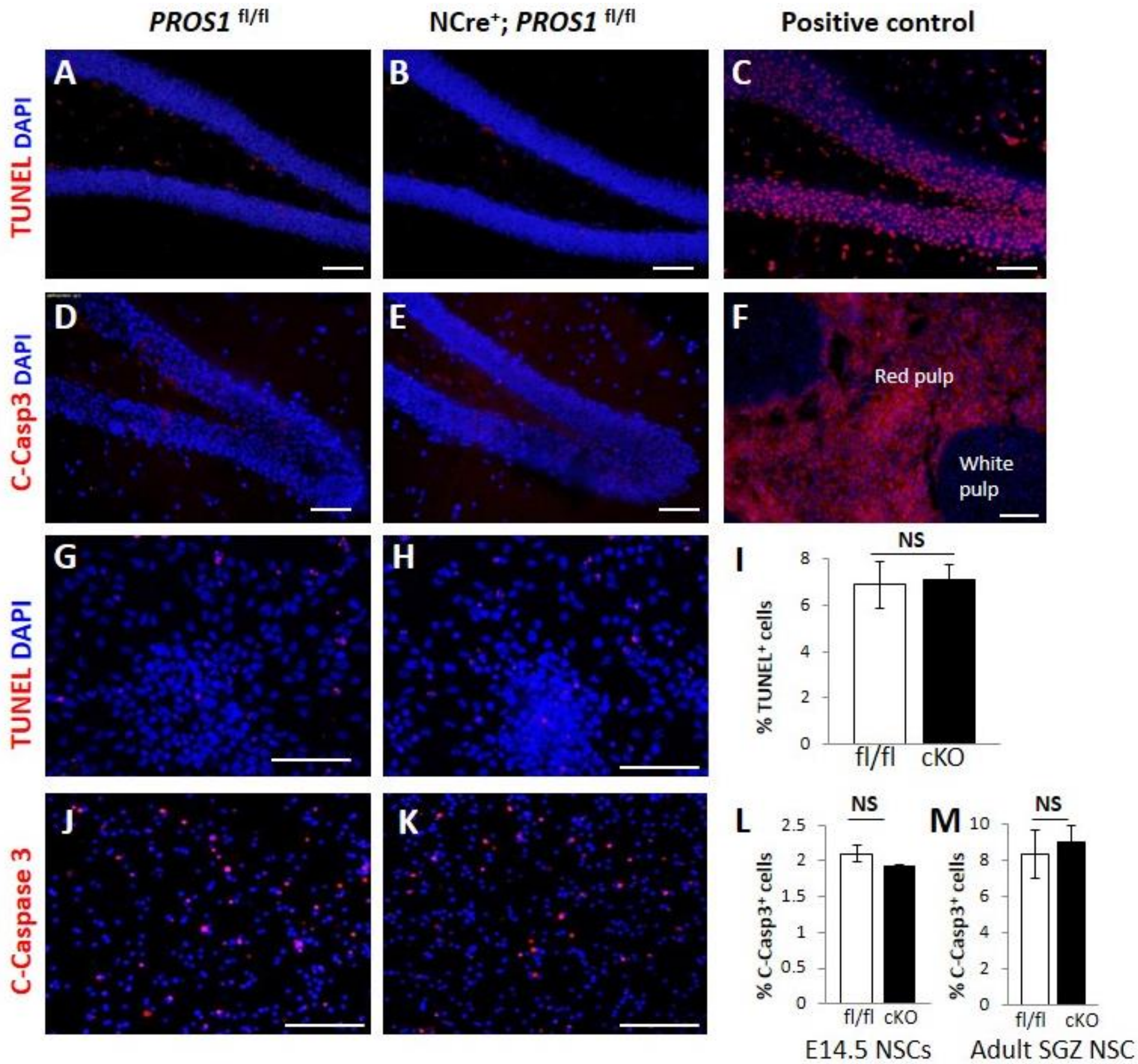

Supplementary Figure 4. No detected increase in cell death following Pros1 deletion.

(A-C) Representative confocal images of hippocampal brain sections subject to TUNEL analysis, identifying apoptotic cells (red) from control (A) and *Pros1*-cKO (B) brains. (C) Positive control for the TUNEL assay following incubation with DNaseI. (D-E) Representative confocal images of hippocampal brain sections stained for cleaved Caspase 3 (C-Casp3), identifying apoptotic cells (red) from control (D) and *Pros1*-cKO (E) brains. (F) A spleen section was used as a positive control for C-Casp3 staining. (G-L) Representative images of neurospheres isolated from control (G, J) and *Pros1*-cKO (H, K) E14.5 embryos and cultured for 10 days, assayed for apoptosis by TUNEL (G-H) or for C-Casp3 immunoreactivity (J-K). (I, L) Quantification of TUNEL-positive (I) and C-Casp3-positive cells in neurospheres isolated from control and *Pros1*-cKO E14.5 embryos (L) and adult SGZ (M). NS = no statistical significance. The mean values and standard errors for TUNEL analysis were derived from 5 individual experiments and from 6 individual experiments for C-Caspase-3. A minimum of 3 and up to 5 embryos per genotype were pooled in each experiment. Scale bars: 50 µm.

1 Supplementary Tables:

Supplementary Table 1: **List of antibodies used**

**Primary antibody**	**Antibody Dilutions**	**Manufacturer**	**Cat. number**
Rat anti BrdU	1:200	AbD serotec	OBT0030
Rabbit anti Protein S	1:100	Millipore	AB15928
Rabbit anti Protein S	1:100	DAKO	A0384
Goat anti GFP	1:200	Abcam	Ab6673
Goat anti Sox2	1:100	Santa Cruz	SC-17320
Mouse anti Vimentin	1:100	Millipore	MAB3400
Mousr anti Nestin	1:100	Millipore	MAB353
Goat anti GFAP	1:400	Cell Signaling	3670
Mouse anti NeuN	1:300	Millipore	MAB377
Rabbit anti Cleaved Caspase-3	1:300	Cell Signaling	9661
Goat anti Actin	1:1000	Santa Cruz	SC-1616
Rabbit anti GAPDH	1:1000	Cell Signaling	2118 (14C10)

Secondary antibody	Antibody Dilutions	Manufacturer	Cat. number
Cy3-Donkey anti Rabbit	1:100	Jackson	711-165-152
Cy2-Donkey anti Rabbit	1:100	Abcam	Ab96919
Cy3-Donkey anti Mouse	1:100	Jackson	715-165-151
Cy2-Donkey anti Mouse	1:100	Jackson	715-545-151
Cy3-Donkey anti Goat	1:100	Jackson	705-165-147
Cy2-Donkey anti Goat	1:100	Jackson	705-545-147
HRP-Goat anti Mouse	1:10,000	Abcam	Ab97040
HRP-Goat anti Rabbit	1:10,000	Abcam	Ab97080
HRP-Donkey anti Goat	1:10,000	Abcam	Ab7125

Supplementary Table 2: **List of primers used**

**Primers for RT-qPCR**		
**gene**	**Forward primer**	**Reverse primer**
mPros1	GCA CAG TGC CCT TTG CCT	CAA ATA CCA CAA TAT CCT GAG ACG TT
mGapdh	AGT TGG GAT AGG GCC TCT CTT	TCC CAC TCT TCC ACC TTC GA
mBMI-1	AAA TCC CCA CTT AAT GTG TGT CC	GGC ATC AAT GAA GTA CCC TCC A
mp16-Ink4a	GAA CTC TTT CGG TCG TAC CC	CGA ATC TGC ACC GTA GTT GA
mp19-Arf	GCC GCA CCG GAA TCC T	TTG AGC AGA AGA GCT GCT ACG T
mNanog	GAA ATC CCT TCC CTC GCC ATC	CTC AGT AGC AGA CCC TTG TAA GC
**Primers used for genotyping**		
gene	**Forward primer**	**Reverse primer**
Cre	ATT TGC CTG CAT TAC CGG TC	ATC AAC GTT TTC TTT TCG G
mPROS1	CAA TAC AAG GCA AAG GGA ATG AGG	GAG CTC TCA GAG ACT AAA C
